# Solitary fibrous tumor of the adrenal gland: a case report and review of the literature

**DOI:** 10.3389/fsurg.2024.1363807

**Published:** 2024-06-05

**Authors:** Changjie Shi, Xiuquan Shi, Ding Wu, Ying Zhang, Dian Fu, Xiaofeng Xu, Wen Cheng

**Affiliations:** ^1^ Department of Urology, Nanjing Jinling Hospital, Affiliated Hospital of Medical School, Nanjing University, Nanjing, Jiangsu, China; ^2^ Department of Urology, Jinling Clinical Medical College of Nanjing Medical University, Nanjing, Jiangsu, China

**Keywords:** solitary fibrous tumor, adrenal tumor, computed tomography, diagnosis, adrenalectomy

## Abstract

Solitary fibrous tumor (SFT) is a rare mesenchymal tumor, probably of fibroblastic origin, mainly in the extremities and pleura. Primary SFT of the adrenal gland is clinically more rare. Here, we report the case of a 47-year-old woman who detected a left adrenal mass on physical examination, without any symptoms, and no laboratory abnormalities. A computed tomography (CT) examination of the adrenal gland suggested a round-like soft tissue density shadow in the left adrenal area. An unenhanced scan showed uneven density of the mass, with a scattered circular-like cystic low-density shadow inside, and an enhanced scan showed obvious uneven enhancement. We considered it to be adrenal pheochromocytoma. Ultimately, the patient was treated with laparoscopic left adrenalectomy. A pathological examination suggested an adrenal SFT. We reviewed previous case reports of adrenal SFTs and summarized the clinical characteristics of adrenal SFT combined with the relevant literature. For adrenal tumors with uneven low-density shadow and uneven CT enhancement features, we should consider the differential diagnosis of adrenal SFT.

## Background

Solitary fibrous tumor (SFT) is a rare spindle cell tumor derived from mesenchymal tissue, accounting for 2% of all soft tissue tumors (1). First reported by Klemperer and Robin in 1931 as a primary pleural neoplasm, SFT mostly originates from the pleura, with cases also reported in the urogenital system, such as the kidney, prostate, and bladder (2, 3). While SFT can occur in endocrine organs, such as the thyroid gland, pancreas, and pituitary gland, primary occurrence in the adrenal gland is even rarer (4–6). A review of the medical literature revealed only 14 cases of adrenal SFTs and one cohort study consisting of 9 cases. This study presents a case of rare adrenal SFT in a 47-year-old woman and provides an overview of previous case reports along with a summary of clinical characteristics combined with relevant literature.

## Case report

**Figure 1:**
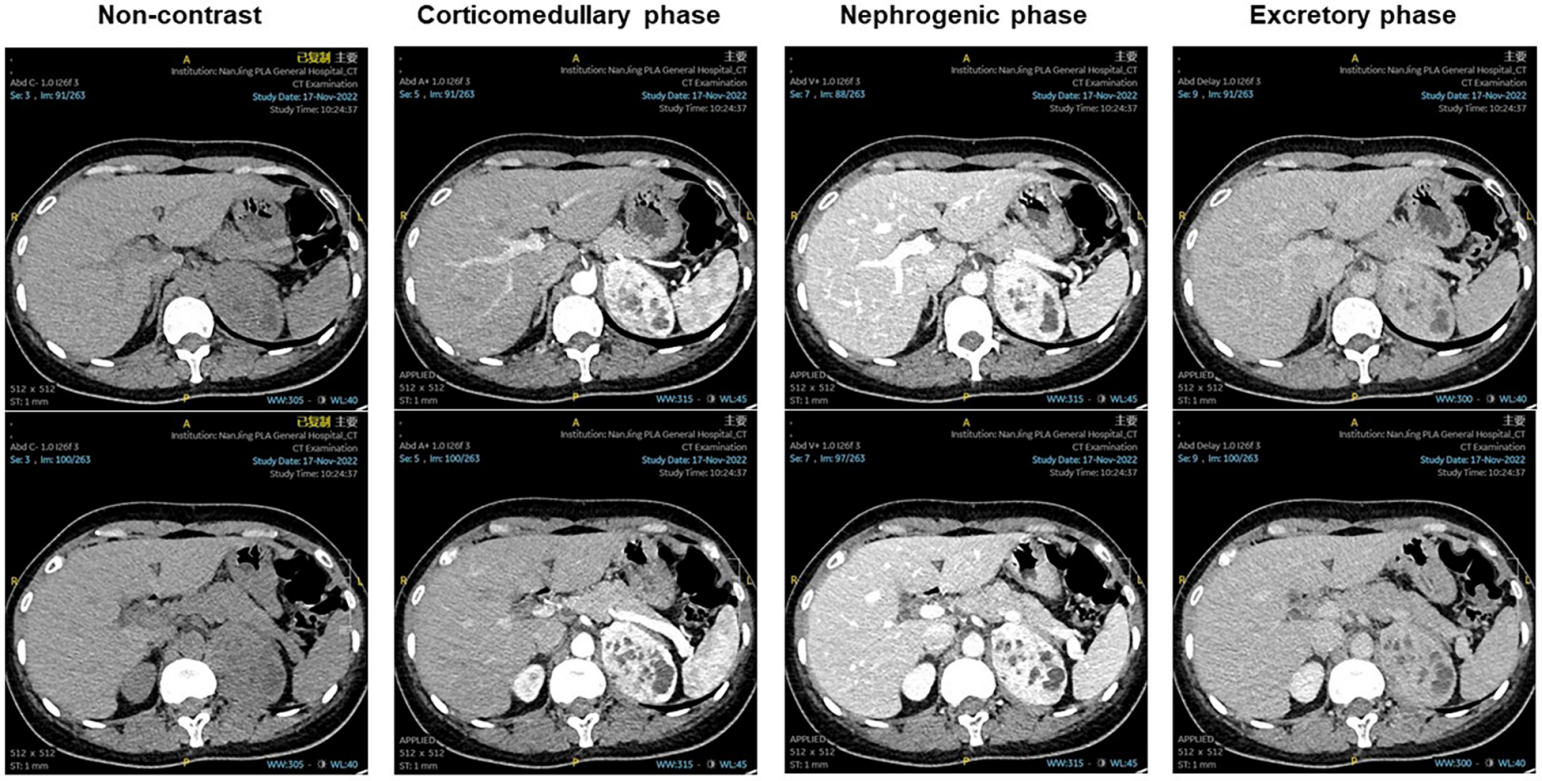
Non-contrast: a round-like soft tissue density shadow in the left adrenal area, with a scattered circular-like cystic low-density shadow inside. Corticomedullary phase, nephrogenic phase, and excretory phase showed obvious uneven enhancement, with scattered circular-like cystic low-density shadow inside.

**Figure 2:**
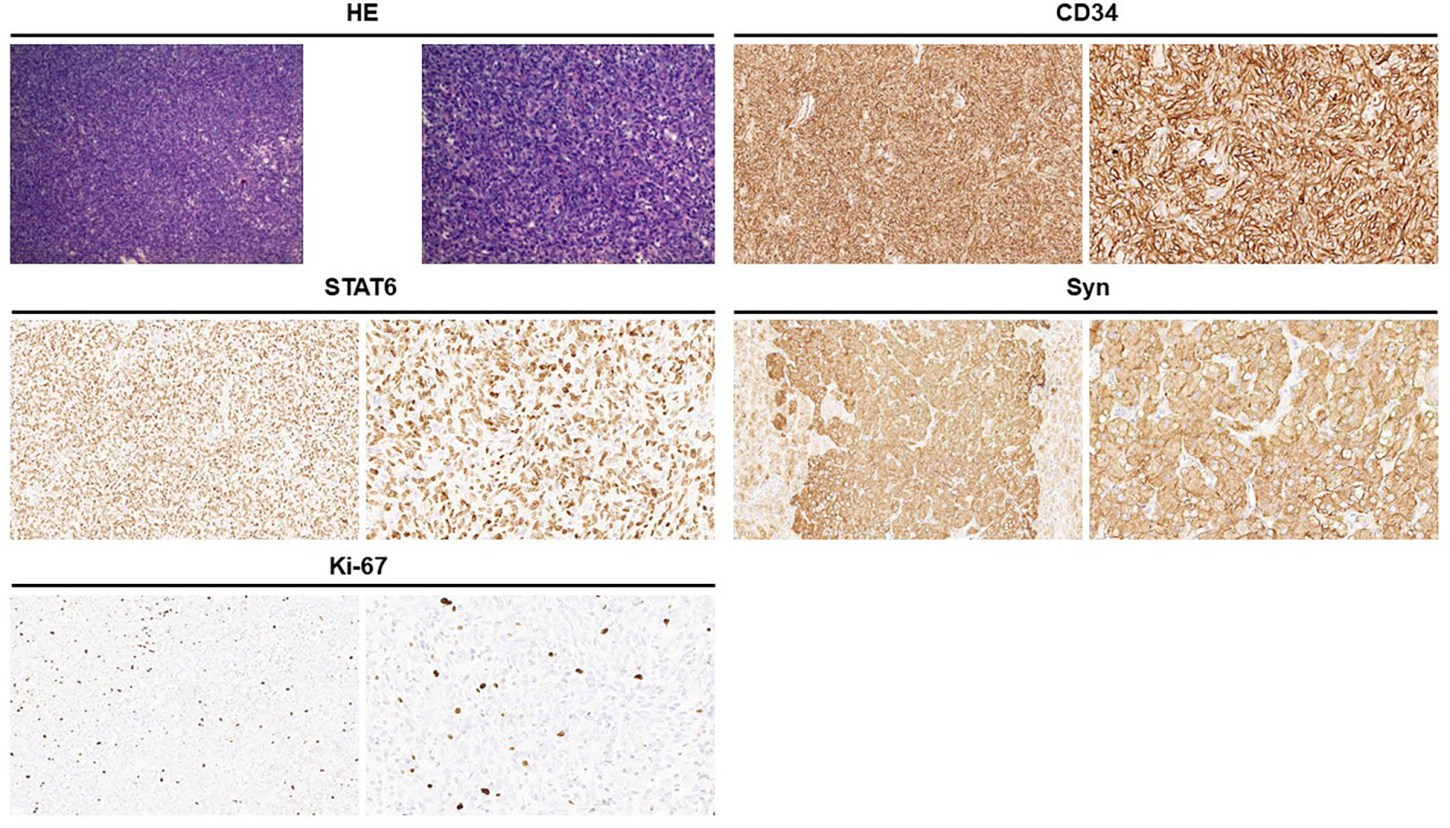
Hematoxylin and eosin staining showed that the tumor cells are spindle-shaped and are distributed in a sheet pattern, with blood vessels (left, ×100; right, ×200). Immunohistochemical staining: CD34 positive (3+); STAT6 positive (3+); Syn positive (1+); Ki-67 is approximately 5% (+).

A 47-year-old woman presented to the urology department with a left adrenal mass detected during a physical examination. The patient did not exhibit symptoms of abdominal pain, hypertension, dizziness, headache, or fever throughout the course of the disease. Upon physical examination, no lump was palpated in the left upper abdomen, and percussion pain in the left renal area was absent. Adrenal-related hormone levels, including plasma cortisol, aldosterone, and catecholamine, were within normal ranges. In addition, other test results showed no significant abnormalities. A computed tomography (CT) examination revealed a round-like soft tissue density shadow in the left adrenal area measuring approximately 6.9 cm × 4.7cm × 5.9 cm. An unenhanced scan displayed uneven density with scattered circular-like cystic low-density shadows inside, while an enhanced scan showed obvious uneven enhancement suggestive of pheochromocytoma (PHEO) (Figure 1). Due to the large size of the tumor and clear surgical indications, magnetic resonance imaging (MRI) was deemed unnecessary for further evaluation. Subsequently, the patient underwent laparoscopic left adrenalectomy after completing preoperative preparation. The removed mass measured approximately 6 cm × 4 cm × 4 cm and exhibited gray and white coloration with focal cystic changes and clear boundaries. A postoperative pathological examination indicated a spindle cell tumor with the immunohistochemistry (IHC) panel showing CD34(3+), STAT6(3+), Synaptophysin (Syn; 1+), CgA(−), S-100(−), desmin(−), SMA(−), SOX10(−), Alpha-inhibin(−), and Ki-67 at approximately 5% (+) (Figure 2). Finally, the patient was diagnosed with an adrenal solitary fibrous tumor (intermediate type). The patient was discharged on postoperative day 5 and was generally in good condition. The ultrasound and laboratory examination in the third month showed no significant abnormal results. After that, we regularly followed the patient for 8 months with no tumor recurrence.

## Discussion

SFTs mostly originate from the pleura, with origin from the adrenal gland being very rare. According to a search of the medical literature, there are only 14 reported cases of adrenal SFT (Table 1) and one cohort study of 9 cases of adrenal SFT (Table 2). Of all the reported cases of adrenal SFT, 15 were in male patients and 8 were in female patients, indicating a higher incidence in men than in women, at a ratio of approximately 2:1. The age range of patients was 13–77 years (mean age 47 years). The clinical manifestations of adrenal SFT are related to tumor volume; larger tumors that compress surrounding tissues or organs can produce symptoms such as back pain or abdominal pain (13 cases), fever (3 cases), hypertension (2 cases), anemia (1 case), elevated cortisol levels (1 case), and paraneoplastic syndrome presenting with symptoms of hypoglycemia (1 case). Our patient had no symptoms and the SFT was detected during a physical examination. Among the reported cases, there were tumors found in the left adrenal gland in 12 cases, right adrenal gland in 10 cases, and both glands in 1 case, suggesting no significant difference between occurrence on either side of the adrenal gland. Tumor sizes ranged from 22 cm × 17 cm × 20 cm to 2.3 cm × 2.5 cm × 3.0 cm. Our patient's tumor measured 6 cm × 4 cm × 4 cm.

**Table 1:** Reported cases with SFT diagnosed as adrenal tumor (*n* = 14).

	Age	Sex	Symptom	Location	Tumor size (cm)	Treatment	F/U (months)	Recurrence during F/U	Positive immunohistochemical staining
1 (7)	71	M	Abdominal pain, blood glucose <50 mg/dl	Left	22 × 17 × 20	Surgery	NM	NM	Vimentin, CD99, Bcl-2, STAT6, CD34
2 (8)	28	M	Mild left abdominal and back pain	Left	6 × 6 × 7	Surgery	24	No	Vimentin, CD34, Ki-67 (5%)
3 (9)	39	F	General fatigue, mild anemia	Left	10	Surgery	20	No	CD34
4 (10)	23	F	Serum and urinary cortisol levels were increased	Left	9 × 6	Surgery	NM	NM	Vimentin, CD34, Bcl-2, MIC-2, Ki-67 (6%)
5 (11)	13	F	Abdominal mass, dull pain, and low-grade fever	Right	18 × 15 × 12	Surgery	3	No	NM
6 (12)	71	M	Physical examination	Right	15.5 × 11.5 × 8	Surgery	NM	NM	Cytokeratin AE1/3, calponin, S-100, CD34
7 (13)	62	F	Back pain	Left	10	Surgery	24	No	CD34, STAT6
8 (14)	77	M	No symptoms	Right	4.5 × 3.4	Surgery	NM	No	STAT6, CD34, Bcl-2
9 (15)	54	M	Hypertension	Both	Right:15 Left:4	Surgery	18	No	CD34, vimentin, Bcl-2, Ki-67 (<10%)
10 (16)	33	M	Fever	Right	2.3 × 2.5 × 3.0	Surgery	NM	NM	CD34, CD99, Bcl-2
11 (17)	52	M	Right lumbar pain	Right	11 × 10 × 9	Surgery	36	No	CD34, Actine
12 (18)	33	F	Right upper abdominal swelling pain and mass	Right	14 × 12 × 6	Surgery + chemotherapy	36	Recurrence	CD34, Vimentin, Bcl-2, CD99, Ki-67 (30%)
13 (19)	37	F	Abdominal pain, excessive sweating	Left	7 × 6	Surgery	NM	NM	NM
14 (20)	50	M	Left lumbar abdomen swelling pain	Left	8	Surgery + chemotherapy	21	Dead	Vimentin, CD99, PDGFR-α, PDGFR-β

	CT	Gross appearance	Microscopic features
1	Unenhanced: low-density shadow	NM	NM
2	Unenhanced: low-density shadow; enhanced: uneven enhancement	Clear boundary, section: gray-brown, soft	The cells are arranged in vortex and bundles, consisting of fusiform cells and oval cells, there are numerous blood vessels
3	Enhanced: uneven enhancement	Clear boundary	The tumor consists of spindle or oval cell proliferation with a laminar pattern
4	NM	Clear boundary, even gray-white fibrous	Irregular arrangement of spindle cells, with large numbers of blood vessels
5	Enhanced: uneven enhancement, calcification	NM	The spindle cells and oval cells showed no pattern of proliferation and contained local blood vessels
6	NM	Section: light pink, partially myxoid	The spindle cells are laminar, bundled and irregularly distributed with “vascular epithelial cells"
7	Enhanced: uneven mild enhancement	Clear boundary, section: yellowish-white, multinodular	NM
8	Enhanced: uneven mild enhancement	Clear boundary, section: gray-white	Spindle cell tumors, and the blood vessels were arranged in a hemangiopericytoma-like pattern
9	NM	NM	NM
10	NM	Solid mass	Spindle cell hyperplasia
11	Unenhanced: uneven low density; enhanced: uneven enhancement	NM	The spindle cells showed a short bundle, layered and irregular distribution
12	Unenhanced: low-density shadow; enhanced: uneven mild enhancement	Section: gray-white, necrosis	Tumor cells are spindle shape, the state is not clear and diffuse hyperplasia
13	Unenhanced: multilocular cystic low-density zone; enhanced: mild enhancement	Cystic solid	The tumor tissue is arranged in a intermanner
14	NM	NM	NM

M, male; F, female; NM, not mentioned; F/U, follow-up.

**Table 2:** Summary of the patients with adrenal solitary fibrous tumor in cohort (21).

Case	Age	Sex	Laterality	Maximum dimension of the tumor (cm)	Cell type	CD34	STAT6	Molecular profile	Treatment	Follow-up (months)	Vital status
1	46	M	Left	7	Spindle	Positive	Positive	ND	Surgery	7	Alive
2	44	M	Right	5	Spindle	Positive	Positive	NAB2:STAT6 rearrangement	Surgery	10	Alive
3	70	M	Left	2.5	Spindle	Positive	Positive	ND	Surgery	23	Alive
4	61	M	Left	3	Spindle	Positive	Positive	NAB2:STAT6 rearrangement	Surgery	13	Alive
5	55	M	Right	2	Spindle	Positive	Positive	ND	Surgery	LTF	LTF
6	64	M	Left	5	Spindle	Positive	Positive	ND	Surgery	29	Alive
7	27	F	Right	7	Spindle	Positive	Positive	ND	Surgery	LTF	LTF
8	58	F	Right	10.5	Spindle	Positive	Positive	ND	Surgery	7	Alive
9	19	M	Left	4	Spindle	Positive	Positive	NAB2:STAT6 rearrangement	Surgery	9	Alive

LTF, lost to follow-up; ND, not done.

On unenhanced CT scans, the SFT typically presents as a round soft tissue density with well-defined borders, and its density is correlated with the amount of collagen fibers in the tissue. On enhanced scans, the SFT shows moderate to high levels of enhancement, characterized by uneven enhancement, possibly due to necrosis and hemorrhage within the tumor (3). MRI reveals predominantly low to moderate signal intensity on T1-weighted imaging (T1WI) and T2-weighted imaging (T2WI), which may be attributed to the presence of collagen fibers within the mass. Kim et al. reported that the signal intensity on T2WI decreases with an increase in collagen components (22). In previously reported cases, one case mentioned uniformly low signal intensity on T1WI and unevenly high and moderate signal intensity on T2WI for adrenal SFT, while two cases noted uneven enhancement during enhanced MRI scans. In our case, the unenhanced CT scan revealed a round soft tissue density shadow with irregular density, including scattered circular cystic low-density shadows; the enhanced scan showed marked irregular enhancement.

Adrenal SFTs require a differential diagnosis, including adrenal PHEO, adrenal cortical carcinoma (ACC), and adrenal metastatic tumor (AMT). PHEO can be qualitatively diagnosed by measuring the concentration of blood-free metanephrines (MNs) or urinary MNs. On unenhanced CT scans, low confounding density is dominant, with some patients showing increased density due to bleeding or calcification. Enhanced CT scans show evident enhancement due to rich blood supply. MRI typically shows mixed signals, with a low signal in T1WI and a high signal in T2WI, significantly enhancing after an enhanced MRI scan. ACC is a rare malignant tumor originating from the adrenal cortex, with 50%–70% exhibiting endocrine function. Unenhanced CT scans show uneven density with irregular, oval, and lobulated shapes as well as cystic changes, necrosis, hemorrhage, and calcification in the lesions. Enhanced scans show irregular ring enhancement. ACC usually invades organs, tissues, or distant metastasis (23, 24). The main common primary tumors of AMT are lung cancer, breast cancer, colon cancer, and thyroid cancer. The CT examination shows diversity; when the tumor is small, it has smooth edges and low uniform density, while larger tumors appear lobulated with blurred edges and uneven density. AMT also exhibits uneven enhancement on both CT and MRI enhanced scans (25).

Adrenal SFTs can be definitively diagnosed through pathological examination and immunohistochemistry. The tumor typically presents with a clear boundary, appearing mostly gray and white in sections, with some instances of mucoid and bleeding necrosis. It has medium hardness, which is related to collagen content. Microscopically, the tumor is characterized by alternating regions of densely packed cells and sparsely distributed cells. The tumor cells themselves are short, spindle-shaped, round, or oval, with abundant red cytoplasm and round, oval, or short spindle nuclei. They are arranged in layers or sheets within the tumor, which also exhibits a rich vascular network forming a typical “hemangiopericytoma-like” area, often containing collagen fibers of varying thickness and shape (26). The microscopic findings from reported cases align with this description. While no single marker demonstrates absolute specificity for SFT diagnosis, combined expression of CD34 and Bcl-2 strongly supports the diagnosis of SFT (10). STAT6 protein expression is highly sensitive and specific for SFT; approximately 90% of SFT cases show positive STAT6 protein expression (27, 28). Immunohistochemical analysis from literature reports indicates a 92% positivity rate for CD34, 50% for Bcl-2, and 50% for Vimentin but only 25% for STAT6 (possibly due to absence of STAT6 protein immunohistochemistry). However, another cohort study showed 100% positive results for both CD34 and STAT6. In our case study, immunohistochemical analysis revealed strong positivity for CD34 (3+), STAT6 (3+), as well as weak positivity for Synaptophysin (1+).

Mosquera and Fletcher suggested that the positive rate of CD34 is associated with tumor differentiation. In general, positive expression of CD34 is high in morphologically benign regions, while the expression of CD34 is often decreased or absent in obvious interchanging regions (29). Bishop et al. suggested that Bcl-2 is more sensitive than CD34 in diagnosing malignant SFTs and that negative Bcl-2 is closely related to the high potential for the deterioration of extra-thoracic SFTs (30). Ki-67 serves as a marker of tumor proliferation, and its increased expression level indicates susceptibility to invasion and metastasis. In the case series by Hanau and Miettinen, the positive rate of Ki-67 was lower in all benign SFTs, with a value of 0%–2%, while histologically malignant SFTs showed a higher positive rate (mean 30%, range 20%–40%) (31). Studies have shown that SFT is characterized by reverse rearrangement mutation in 12q13-5 to produce a fusion of NAB2-STAT6 gene, leading to overexpression of STAT6 protein. NAB2-STAT6 gene fusion is considered to be a molecular marker for SFT (three cases in Table 2 underwent genetic testing and all showed NAB2-STAT6). The most common are the NAB2 exon 4—STAT6 exon 3 and NAB2 exon 6—STAT6 exon 16/17 conjunctions. The second most common NAB2-STAT6 genotype is the NAB2 exon 6—STAT6 exon 16/17 conjunction, which is associated with more aggressive clinicopathologic characteristics. This genotype/phenotype variant mostly occurs in extra-thoracic SFTs and mainly affects young patients. STAT6 plays an important role as an immunohistochemical marker for distinguishing SFTs, with a sensitivity of 98% and specificity of nearly100% (32, 33). Therefore, for adrenal SFTs, NAB2-STAT6 gene detection should be performed to increase diagnostic accuracy, assess aggressiveness, guide treatment decisions, and predict prognosis.

Currently, complete surgical resection of the tumor is considered the optimal treatment for adrenal SFTs, with resectability being the key prognostic indicator. Complete resection significantly reduces the recurrence rate of SFTs, although malignant SFTs have a higher recurrence rate compared to cases of benign SFTs (34). The diagnostic criteria for malignant SFTs include the following: (1) abundant and dense cells; (2) cell pleomorphism; (3) nuclear fission elephant ≥4/10 HP; and (4) necrosis and hemorrhage. Tumor edge infiltration also holds significance. Reported cases have mainly undergone complete surgical resection, with postoperative follow-up in the range of 3–36 months showing no tumor recurrence or metastasis in cases of benign adrenal SFTs. In two cases of diagnosed malignant adrenal SFTs, postoperative adjuvant chemotherapy was used to reduce tumor recurrence and metastasis. One case experienced tumor recurrence 36 months after surgery but showed no further recurrence or metastasis after a second surgery at 18 months. The other patient tested positive for platelet-derived growth factor receptors-α (PDGFR-α) and platelet-derived growth factor receptors-β (PDGFR-β) and received oral imatinib mesylate treatment but unfortunately died due to tumor metastasis after 21 months of follow-up. Our patient was classified as having intermediate adrenal SFT and showed no signs of tumor recurrence during an 8-month postoperative follow-up period. Most adrenal SFTs demonstrate benign or intermediate biological behavior, resulting in a good prognosis with rare occurrences of malignancy in this context.

In conclusion, when dealing with adrenal tumors, it is important to consider the possibility of an adrenal SFT diagnosis if an unenhanced CT scan reveals a low-density shadow and an enhanced scan shows uneven enhancement. The definitive diagnosis of adrenal SFT relies heavily on pathological examination and immunohistochemistry. At present, there is no established standard treatment plan, with surgical resection remaining the primary method. The prognosis for cases of benign or intermediate adrenal SFTs is generally favorable; however, cases of invasive and malignant adrenal SFTs are more prone to distant metastasis and require close monitoring and review. Targeted therapy has emerged as a new therapeutic approach in recent years, showing promise for eligible patients; nevertheless, its clinical efficacy still lacks substantial research support. Large-scale research and treatment guidelines for adrenal SFTs are currently lacking, indicating the need for further investigation in this area.

## Data availability statement

The original contributions presented in the study are included in the article/supplementary material, further inquiries can be directed to the corresponding authors.

## Ethics statement

Written informed consent was obtained from the individual(s) for the publication of any potentially identifiable images or data included in this article. Written informed consent was obtained from the participant/patient(s) for the publication of this case report.
